# Case management of ultra-processed food addiction: a theoretical perspective

**DOI:** 10.3389/fpubh.2026.1814936

**Published:** 2026-05-19

**Authors:** Octavian Vasiliu

**Affiliations:** 1 Discipline of Psychiatry II, Department of Clinical Neurosciences, Carol Davila University of Medicine and Pharmacy, Bucharest, Romania; 2 Department of Psychiatry, Dr. Carol Davila University Emergency Central Military Hospital, Bucharest, Romania

**Keywords:** behavioral addictions, eating disorders, food addiction, metabolic dysfunctions, RDoC model, stepped care model, substance use disorders, ultra-processed foods

## Abstract

Ultraprocessed food addiction (UPFA) is a complex and conceptually contested construct, uniting clinical and pathophysiological elements of eating disorders (EDs), behavioral addictions (BAs), substance use disorders (SUDs), and metabolic disorders (MDs). Despite increasing empirical interest, no formal diagnostic criteria or structured treatment recommendations currently exist. The case management of individuals presenting with UPFA raises important challenges for mental health specialists, as no therapeutic guidelines for this condition exist. This theoretical perspective proposes a stepped-care model, including psychotherapeutic, nutritional, and pharmacotherapeutic interventions, as well as lifestyle changes, based on a Research Domain Criteria (RDoC) framework. By mapping UPFA components onto positive and negative valence systems, cognitive control processes, social mechanisms, arousal regulation, and habit circuitry, the suggested model conceptualizes this addiction as a dimensional pattern of dysregulated reward processing, stress responsivity, impaired executive control, and automatized eating behaviors. Although most recommendations are extrapolated from studies involving patients with EDs (mainly binge eating disorder and bulimia nervosa), substance use disorders (SUDs), and MD (i.e., obesity), the proposed framework emphasizes mechanism-based assessment, individualized treatment formulation, and empirical testability. This perspective aims to provide a structured foundation for clinical decision-making while advancing a cautious research agenda to clarify the validity, boundaries, and therapeutic implications of UPFA.

## 1 Introduction

Ultra-processed food addiction (UPFA) is situated at the intersection of substance use disorders (SUDs), behavioral addictions (BAs), eating disorders (EDs), and metabolic disorders (MDs), with a high degree of clinical overlap with all these pathologies. While some pathogenic models emphasize the addictive potential of specific nutrients, given the highly engineered and reinforcing properties of ultra-processed foods, aligning this condition with SUDs, other perspectives conceptualize food addiction as a BA, characterized by impaired control, cue-reactivity, habit formation, and reinforcement learning processes, and still other paradigms promote an integrative concept (1–3). Regarding UPFA as a SUD, craving for specific UPF, with loss of control over their use, and withdrawal-like symptoms, as well as continued consumption of such foods, although there are negative consequences for one’s own health, incapacity to cut down the use of UPF, and even tolerance have been reported in relation to this addiction (4–8). Considering UPFA as an SUD opens therapeutic options such as abstinence-focused strategies, motivational interviewing, or harm reduction (9, 10). Viewing UPFA through the lens of a BA has important implications for case formulation, as it shifts the focus toward mechanisms such as reinforcement learning, compulsivity, and executive dysfunction. From a clinical perspective, framing UPFA as a BA supports the use of interventions targeting cue-reactivity, craving management, habit reversal, and cognitive control, rather than relying exclusively on models derived from substance exposure (11, 12). In the present paper, an integrative perspective on the UPFA is preferred due to its theoretical and practical fecundity, as it can stimulate research across psychosocial, neurobiological, and pharmacological domains. It is therefore expected that a focus on the pathogenic mechanisms and potential directions for developing empirical studies, starting from vulnerability and environmental factors, would be more useful for increasing the interest of researchers and clinicians alike in this pathology, rather than adhering to a categorical approach.

When UPFA is explored in relation to EDs, episodes of binge eating and eating behaviors influenced by emotional states have to be considered (4–8, 13). Supporting this observation, the prevalence of food addiction was calculated to be 55% in individuals with a clinical diagnosis of binge eating, according to a meta-analysis (*n* = 272 studies) (14). A careful assessment is required to differentiate UPFA from EDs and to identify cases where both conditions co-occur, as this distinction has important implications for therapeutic management, evaluation of prognosis and course, and formulation of the most appropriate monitoring plan. Also, insulin resistance, leptin resistance, and dyslipidemia may be common in patients with metabolic disorders and UPFA, a problem that adds to the common risk factors of the two pathologies and the high rate of co-occurrence of UPFA and obesity (4–8, 13, 15).

Although the concept of “food addiction” was mentioned for the first time in the medical literature by Randolph in 1956, there are data on chocolate addiction starting from the 19th century, more specifically in 1890 (16–18). Psychoanalysts also explored food addiction from the perspective of an oral complex, but those considerations refer to virtually any type of overeating, thus being only marginally relevant for the topic of UPFA (17, 19). The interest of scientists in investigating the pathophysiology and clinical particularities of UPFA increased in the last decade, with research becoming more focused on food addiction as a distinct entity from obesity, bulimia nervosa (BN), and binge eating disorder (BED) (17). Animal studies have been conducted in this field (based on possible similarities between BED and food and drug craving, i.e., a decreased dopamine D2 receptor availability), and neuroimaging results have been provided (e.g., different activation patterns and connectivity in brain reward circuits and hypothalamus), as well as structured scales for evaluation of UPFA symptoms in the general population (e.g., Yale Food Addiction Scale- YFAS, YFAS 2.0, or modified YFAS- mYFAS) (17, 20–25).

After seven decades since its mention in a medical source, food addiction still remains a heterogeneous construct, with evidence from systematic reviews and empirical studies indicating significant variability in prevalence, course trajectories, symptom expression, and comorbidities (26, 27). Inter-individual differences in reward sensitivity, impulsivity, emotional regulation, and other biological and psychological vulnerability factors have been reported in studies exploring food addiction, supporting the idea of addictive eating phenotypes, rather than a unitary diagnostic concept (26, 27). Adding to the heterogeneity of the concept, different subtypes of food addiction have been theorized based on specific food components, with the strongest evidence supporting sugar and sugar–fat combinations, which are able to induce craving, loss of control, and neurobiological changes similar to SUDs (28–30). Chocolate-related craving has also been described, often associated with emotional regulation and reward sensitivity, and salted foods have been explored in a similar context (28–30). Overall, current research suggests that addictive-like eating is primarily driven by ultra-processed, hyperpalatable foods, with individual variability in preference reflecting underlying neurobehavioral differences (28–30).

Important steps have been taken to formulate diagnostic criteria for UPFA, and a Delphi consensus involving 40 clinicians, researchers, and academics from 10 countries, along with a team of facilitators, reached agreement on several key features of UPFA, such as the existence of a unique disorder of UPFA and the resemblance of UPFA to other SUDs (31). The consensus served as the basis for requesting recognition of UPFA as a disorder by the ICD-11 committee within the World Health Organization (WHO) (31). Nevertheless, the lack of clearly, agreed-upon diagnostic criteria negatively interferes with the possibility of conducting large-scale, good-quality trials focused on the therapeutic management of patients with UPFA. Therefore, a model that integrates available data into a therapeutic algorithm for these patients may be a first step toward validating a clinical guideline.

## 2 Multidimensional assessment in ultra-processed food addiction

A biopsychosocial model of assessment for UPFA may be considered most relevant because of the clinical and physiopathological complexity of this type of pathological behavior and the need to incorporate multiple methods tailored to different levels of UPFA severity. Due to the high degree of phenomenological overlap of UPFA with ED and SUD, the Research Domain Criteria (RDoC), a framework developed by the National Institute of Mental Health (NIMH), which is based on dimensions of behavior and neurobiology, rather than purely clinical features, was chosen for a description of this pathology (32, 33). The major domains of RDoC are (1) the positive valence system, including anticipation, obtaining, and responding to rewarding stimuli, (2) the negative valence system, related to responses to aversive stimuli, (3) cognitive systems, referring primarily to executive functioning, (4) systems for social processes, (5) arousal or regulatory systems, and (6) sensori-motor systems (32, 33). Each of these domains includes multiple constructs, representing various degrees of detail that allow for a more nuanced description of the main dimensions, and the two axes, i.e., the neurodevelopmental and the environmental, which are used for incorporating all relevant aspects to the respective condition (32, 33). The application of this model to UPFA is supported by research that evaluated SUDs in adolescents, highlighting the importance of maladaptive emotional reactivity, altered reward sensitivity, and maladaptive habit formation, impulsivity, and limited social networking, low metacognitive abilities, heightened stress and insomnia, corresponding to the main dimensions of RDoC (34). Such research opens a new way of therapeutically addressing the risk factors in vulnerable populations for SUDs, and, at the same time, enhancing the protective factors in the same groups (such as adaptive emotional regulation, healthy resources of rewards, and resilience) (34). When adapted to UPFA, the RDoC framework allows for a subtle characterisation of behavioral and neurobiological dimensions of this condition: (1) the positive valence system is represented by the reward processes, which stand as the core of the UPFA, with relevant constructs as reward valuation, reward learning, and habit formation (35–43); (2) the negative valence system is represented by stress and aversive states, and its core constructs are acute threat, sustained threat, loss, and frustrative nonreward (44–47); (3) cognitive systems involved in this case are executive control and self-regulation, with constructs such as cognitive control, response inhibition, and working memory (48–52); (4) relevant concepts for social processes in UPFA are social communication, self perception, and affiliation and attachment (4–8); (5) arousal/regulatory systems are relevant for UPFA due to the involvement of concepts such as arousal, circadian rhythms, and sleep–wake systems (53–55); (6) the sensorimotor system becomes important for UPFA through automatic eating behaviors, cue-triggered motor routines, and loss of voluntary control over eating sequences (56–61).

The neurobiology of the *positive valence system* in the case of UPFA is based on dopamine signaling in the nucleus accumbens and the ventral tegmental area, hyperresponsivity to highly palatable foods, and craving triggered by olfactory and visual stimuli, thus paralleling SUDs in this domain (35–43). The *negative valence system* fits within the UPFA conceptualisation from the RDoC perspective, supported by neuropsychological arguments such as emotional regulation through food, withdrawal-like irritability, and eating to reduce negative mood (44–47). C*ognitive systems* are important in UPFA due to reduced inhibitory control around food cues, impaired decision-making under emotional stress, and difficulty in delaying gratification, all of which may be linked to reduced prefrontal regulation over reward systems (48–52). The *arousal/regulatory systems* correspond to disrupted hunger-satiety signaling, stress-hormone dysregulation, sleep deprivation as a way of increasing reward sensitivity, and hormonal influences (ghrelin, leptin) (4–8). The *social processes* correspond to shame and self-concept distortions, social cue-triggered overeating, and cultural reinforcement of ultra-processed food consumption (53–55, 62–66). The *sensorimotor system* includes neural circuitry such as the dorsal striatum (for habit formation), basal ganglia motor loops, motor cortex, and corticostriatal pathways (57–61).

According to the RDoC framework, the evaluation of multiple biological components that support each dimension is required, e.g., genes for D2 dopamine receptors, neurotransmitters (e.g., dopamine or opioids), circuits (mainly the mesolimbic reward pathway), other molecules (e.g., hormones), behaviors (e.g., binge eating), and behavioral assessment (e.g., YFAS scores). This type of scientific endeavour is still in its early stages, but applying a conceptual model to UPFA may increase researchers’ interest in conducting fundamental research focused on the neurobiological, genetic, and metabolic correlates of this addiction.

**Figure 1:**
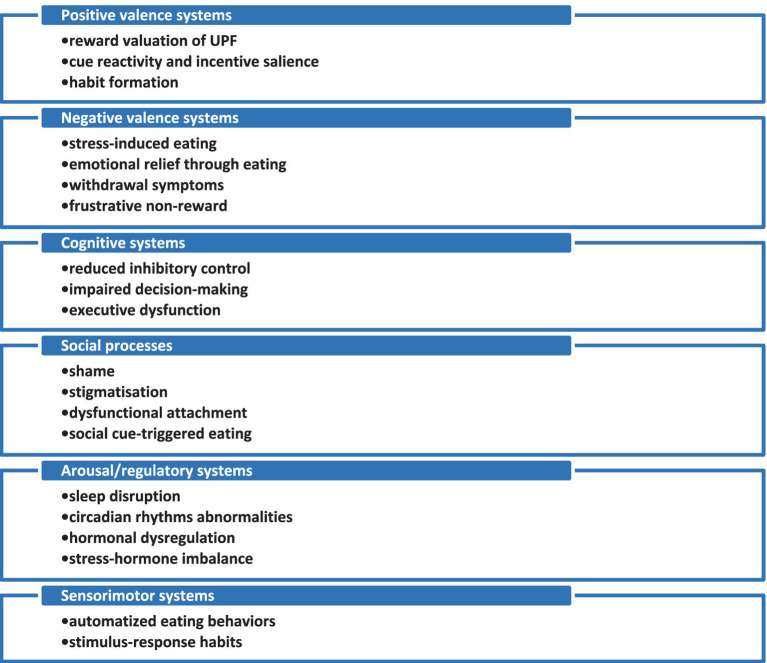
RDoC-based multidimensional model of ultra-processed food addiction. UPF, ultra-processed foods.

Taken together, the current RDoC model provides a nuanced perspective on UPFA, as this pathology may not be regarded as a single categorical diagnosis, but as a dimensional one (Figure 1). This addiction is conceptualised, according to this model, as a consequence of a loop of reward/cue reactivity, weakened control, stress/negative affect relief, dysregulated arousal/sleep/metabolic signals, automatized habits, and social/self processes. An adequate treatment would address each of these components, with the case manager checking the degree of involvement of each element, since their participation in the pathogenesis of each case differs, thus contributing to the heterogeneity of UPFA.

## 3 Therapeutic algorithm for patients with ultra-processed food addiction

Applying the same RDoC epistemological framework to the *case management* of patients diagnosed with UPFA, coordination of a multidisciplinary team is recommended in order to target various biological, social, and psychological dysfunctions.

In this regard, a Collaborative Care Model (CCM) can be adapted for UPFA by extrapolating from chronic conditions such as EDs, obesity, and SUDs (67–71), including a case manager, psychotherapist, clinical psychologist, dietitian/nutritionist, psychiatrist, and endocrinologist. This team would bring together the necessary competencies for a multidimensional approach to therapy and monitoring of individuals presenting with UPFA. Regarding the accurate monitoring needed for these patients, the main outcomes would be, at minimum: (1) body mass index (BMI), waist circumference, lipid profile, and glycemia and glycated haemoglobin for the *biological dimension*; (2) YFAS/YFAS 2.0/mYFAS scores and a weekly log of binge eating episodes for the *psychological dimension*; (3) a quality of life scale (such as EuroQOL 5D 5L), as well as the life activities and participation domains of the World Health Organization Disability Assessment Schedule (WHO-DAS2.0), for the *social dimension*. An initial psychiatric and clinical psychology examination would reduce the risk of leaving psychiatric comorbidities undetected, such as anxiety disorders, depressive disorders, bipolar disorders, trauma and stress-related disorders, ADHD, personality disorders, substance use disorders, eating disorders, and various other behavioral addictions. Also, the endocrinological screening visit would have as its main objective assessing the risk of metabolic dysfunctions, some of which may have vital prognostic significance, such as diabetes.

Specific interventions for UPFA are expected to address all RDoC dimensions that can be therapeutically targeted. *Psychotherapeutic interventions* such as enhanced cognitive behavioral therapy (CBT-E), created by Fairburn (72), may address the loss of control over eating, binge patterns, and overvaluation of weight or body shape. However, UPFA may include patterns without body shape or weight overvaluation, cases that would require CBT-E adaptation. CBT-E contains modules of psychoeducation, techniques of focusing on regular meals and trigger situations, self-monitoring of food and thoughts related to eating, and exploration of body image disturbances (72–74). Interventions created for SUDs would also be useful for patients with UPFA, such as motivational interviewing and addiction-focused CBT, targeting cue identification, increasing insight, training craving management skills, and behavioral coping skills, planning relapse prevention, and cognitive restructuring of addiction beliefs (75–77). Self-help groups may also be effective for patients with UPFA, as they have been shown to be useful in addressing various EDs (78). Adaptation of these interventions for UPFA would require techniques for targeting cue-reactivity to ultra-processed foods, exploring and de-structuring the cycle of cue-craving-consumption-relief-shame, and working with dysfunctional models of attachment and affiliation. An online, peer-supported, psychoeducational intervention for individuals with UPFA (*N* = 86 participants), with a 6-week duration, led to statistically significant improvements at the end of the study and 6-month follow-up, i.e., greater self-awareness, reduced impulsive eating and eating behaviors, as well as increased confidence in managing UPFA symptoms (79). The non-judgmental approach, the promotion of hope in a future beyond the intervention, and the high acceptability were strengths of this program (79).

*Nutritional counselling* refers to non-restrictive, addiction-informed nutrition, with gradual elimination or substitution of UPF, structured meals to stabilize glycemia, prioritization of protein and fiber, and food environment engineering. A Position Statement of the American Dietetic Association (80) shows that nutrition intervention, including nutrition counselling, is essential for patients with EDs during assessment and treatment. Nutrition professionals play a vital role within multidisciplinary eating disorder treatment teams, contributing specialized expertise in nutrition and physiology while supporting behavior change within the broader psycho-socio-cultural context of eating (81). The role of abstinence in the process of recovery in individuals with FA was highlighted by Tarman (9), who explored this phenomenon within the framework of Koob’s addiction recovery model (9, 10). The addiction is a multi-stadial process, and the essential phase, where the addictive substance or behavior becomes central to the individual’s daily life (i.e., “hyperkatifeia”), is characterized by engaging in the problematic activity to avoid negative emotions, rather than to seek pleasure (9, 10). The neurobiology of hyperkatifeia involves dysfunctions in the extended amygdala and its dopamine, enkephalin/endorphin, gamma-aminobutyric acid, and glutamate transmission, all of which mediate a negative hedonic set point (10). In this phase, abstinence is the only action that could prevent further deterioration of the individual’s functionality. Applied to the UPFA, a low-carb keto diet, involving stopping the use of UPF, would correspond to an abstinence-focused intervention, and there are clinical studies supporting this approach (82–84). In a 12-month follow-up of a low-carbohydrate and psychoeducational program for the treatment of UPFA, significant and sustained improvements in the symptoms and mental well-being were observed, using structured evaluation (i.e., YFAS 2.0, ICD-10 symptoms of SUD related to food- CRAVED, and Warwick Edinburgh Mental Wellbeing Scale- WEMWS) (84). Another 6-year retrospective follow-up of an abstinence-based intervention for individuals with FA (*N* = 267) showed that 72% of the participants maintained weight loss of >5%, with a significant association between sustained weight loss and adherence to the abstinence-based food plan (85).

A telemedicine intervention using TOWARD principles (i.e., Text-based communication, Online interactions, Wellness coaching, Asynchronous education and community support, Real-time biofeedback and remote monitoring, and Dietary modification) applied in individuals with food addiction and binge eating was associated with a decrease in both types of symptoms (by 40.7 and 34.7%, respectively) (86). The benefits of such an intervention administered in an employee wellness setting were also observed in body weight, medication-related financial costs, and mental health symptoms related to food (86).

*Harm reduction interventions* may also be applied during the nutrition counseling, if abstinence is not possible due to various cultural (e.g., ritualization of UPF consumption), social (e.g., the use of UPF in certain social contexts), psychological (e.g., the risk of developing negative emotions during UPF use withdrawal and unhealthy relationships with foods), or even economic factors (i.e., UPFs are less expensive and more readily available than their healthier alternatives) (9). Harm reduction approaches suggested for UPFA include a gradual reduction or substitution of sugar intake in the early phases, replacing high-sugar foods with lower-sugar alternatives or sweeteners, and applying mindful eating practices and relaxation techniques to reduce emotional eating (9, 87).

*Pharmacotherapy* is to be considered only as an adjunctive intervention for UPFA, mainly where severe cravings persist despite psychotherapy and nutrition, or comorbid metabolic disorders are identified, such as obesity, diabetes, dyslipidemia, or if repeated relapse is identified. Based on a very limited dataset, naltrexone +/− bupropion, lisdexamphetamine, and topiramate may be useful (88–92). GLP-1 receptor agonists (e.g., liraglutide, semaglutide) may reduce the reward salience of ultra-processed foods, and serotonin selective reuptake inhibitors (SSRIs), mainly fluoxetine and escitalopram, for comorbid anxiety or depressive disorders may be recommended, but data are extrapolated from other EDs or metabolic disorders (88–97).

A stepped-care model for UPFA needs to include several core stages and multiple optional interventions. *Step 1* addresses mild forms of UPFA (YFAS2.0/mYFAS score 2–3), with no significant psychiatric and organic comorbidities, and low levels of functional impairment. Adequate care interventions for this stage would require psychotherapy, mainly motivational interviewing and psychoeducation, with the possibility of accessing CBT-E, nutrition counselling, and lifestyle-changing techniques, such as food environment restructuring. Psychological monitoring, focused on craving intensity, binge frequency, and discomfort related to eating, would orient further interventions. The administration of YFAS/YFAS2.0/mYFAS is recommended at each control visit.

*Step 2* of this algorithm would be recommended in cases of moderate UPFA (YFAS2.0/mYFAS score 4–5) with no significant comorbid conditions, or when step 1 interventions were not effective, as reflected in the monitored outcomes. This stage includes structured psychotherapy, a planned meal regimen, and, if needed, pharmacotherapy. A more intensive monitoring is recommended, including everything mentioned in step 1, as well as metabolic markers, quality of life, and overall functioning.

*Step 3* is reserved for severe cases of UPFA (YFAS2.0/mYFAS≥6), mild/moderate UPFA cases with significant comorbidities, when functional consequences occur across multiple domains of daily life, and when interventions in steps 1 and 2 did not lead to satisfactory outcomes. This stage would necessitate a combination of nutritional counselling, psychotherapy, and pharmacotherapy, with intensive medical and psycho-social monitoring. Although the efficacy of associating psychotherapy and pharmacotherapy was not entirely proven for EDs (98, 99), at least in the case of comorbid organic and/or psychiatric disorders, its recommendation seems justified.

**Figure 2:**
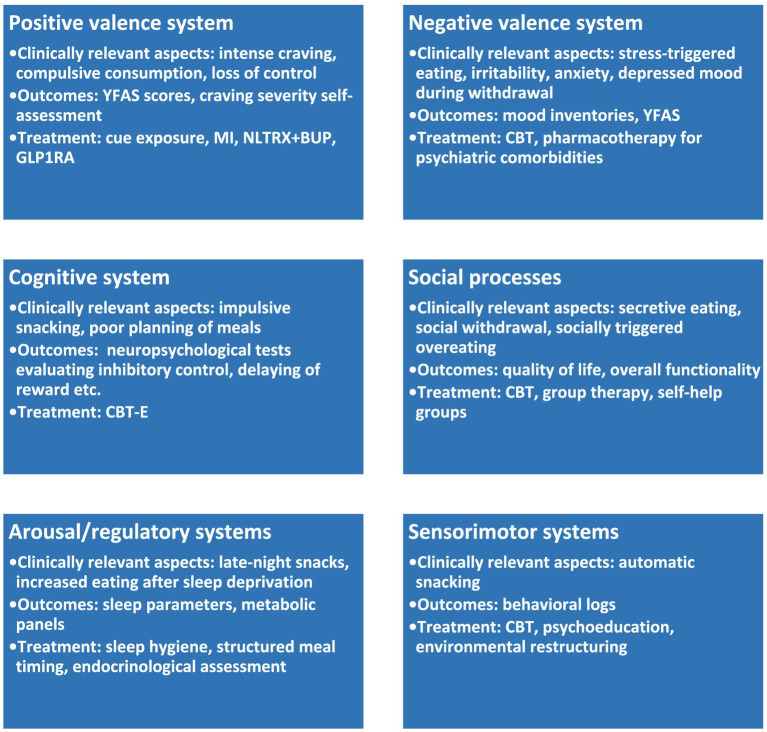
Mechanism-based case management. CBT, cognitive-behavioral therapy; CBT-E, enhanced CBT; YFAS, Yale Food Addiction Scale; MI, motivational interviewing; NLTRX, naltrexone; BUP, bupropion; GLP1RA, glucagon-like peptide 1-receptor agonists.

Applying the RDoC framework to the case formulation of UPFA, the core dimensions of dysfunctions are: (1) positive valence systems- lifestyle-focused interventions and psychoeducation would be helpful by reducing the exposure to high-risk cues by redesigning the environment; psychotherapy would intervene through relaxation techniques and cue exposure with prevention of response; (2) negative valence system- to attain the objective of stopping eating as a stress relieving act, psychotherapy programs should increase the awareness of stressful moments and to train coping skills; psychotherapy and/or pharmacotherapy may also be required to treat comorbid anxiety or mood disorders; (3) cognitive systems- in order to enhance the executive control, CBT interventions are of essence, with training of coping skills, planning of daily activities (eating included), and inhibitory control training; (4) social processes- the goal is to reduce shame related to eating (binge eating, obesity) and to build a supportive network for the patient, and psychotherapy would have to identify dysfunctional cognitive beliefs, automatic thoughts related to eating and body weight/shape overvaluation; self-support groups can also be added to mitigate the negative impact of such cognitive dysfunctions and to relieve the burden of shame and guilt; training of social skills may be added; (5) arousal/regulatory systems- psychotherapy +/− pharmacotherapy may be added to control the sleep pattern; lifestyle interventions are recommended for regular meal timing, to reduce excessive hunger and rebound eating, and also to restrict the access to ultra-processed foods; (6) sensorimotor systems- the interventions should aim at reducing automaticity when eating, to break the stimulus–response habit chain and build new, adaptive behaviors; psychoeducation and simple psychotherapeutic techniques are useful- e.g., when exposed to cues, performing a brief alternative motor routine may help; when out of the house, restrict the visitation of areas with high exposure to sensitive cues; replace “snack loop” with “close the kitchen at a certain hour” ritual; deliberate interruptions mid-episode (set timer, stand up, change location) (Figure 2).

## 4 Discussion

Although the evidence in the field of UPFA treatment is quite limited, it must be kept in mind that this addiction may be only the tip of the iceberg, under which various layers of psychological and psychiatric problems could be detected, ranging from obesity and diabetes to personality disorders and mood disorders. Although UPFA presents features of both behavioral and substance use disorders, it also includes elements of eating disorders and may be associated with consequences across the metabolic disorder spectrum (100–102). While abstinence-based approaches may be relevant in selected cases, particularly where specific ultra-processed foods act as strong triggers, a flexible and individualized strategy is likely to be more appropriate given the heterogeneity of UPFA. Multiple interventions are nevertheless possible, ranging from nutritional counseling to support groups and pharmacotherapy, although they still require more empirical studies to confirm their long-term efficacy. The heterogeneity of UPFA itself makes it harder for the case manager to formulate the most adequate therapeutic plan, since the lack of control over eating, tolerance to desired foods, and negative consequences for physical, mental, and social health, as well as the level of distress, may vary considerably from one individual to another (103–105). To further complicate case management, there are no criteria for a formal diagnosis of UPFA, as this entity is not recognized by contemporary nosographic systems such as ICD-11 and DSM-5 TR, but it follows the same pattern as SUDs (106, 107). The mental health specialist may, however, monitor the evolution of these patients by administering validated scales for UPFA, such as YFAS/YFAS2.0/mYFAS, which have been widely used and demonstrate good psychometric quality, and using a large number of metabolic analyses, and other psychometric outcomes (e.g., mood-related, quality of life, overall functioning) (108).

Framing the UPFA according to the RDoC model offers advantages from both theoretical and practical perspectives. While this type of modelling helps develop new studies to clarify the pathogenesis and potential treatments for UPFA, at a practical level, it could encourage a more structured approach to individualising therapy. The proposed RDoC-informed framework for UPFA generates empirically testable hypotheses while avoiding the conundrum posed by this condition’s nosological status. If UPFA represents a clinically meaningful dimensional construct, individuals reporting high levels of ultra-processed food–related craving and loss of control may exhibit heightened cue-reactivity to ultra-processed foods compared to minimally processed alternatives, alongside measurable alterations in reward-related neural circuitry. However, such findings should be interpreted alongside those for BED and obesity to clarify the extent of overlap and divergence. It is also plausible that UPFA severity correlates more strongly with reward sensitivity, habit formation, and stress-reactivity measures than with weight or shape overvaluation, suggesting differential weighting of RDoC domains across individuals rather than categorical separation.

Approaching complex cases involving UPFA and somatic comorbidities would especially benefit from a structured, stepped-care approach. The model proposed in this article enables such a multidimensional intervention for UPFA, obesity, diabetes, metabolic syndrome, and related conditions. Other authors also suggested a structured approach for this significant subgroup of individuals with UPFA, highlighting the importance of adjusting the interventions to the specifics of each case. Such a suggested strategy incorporating screening (e.g., CRAVED is accessible and easy to use), dietary interventions (i.e., addiction-informed nutritional support), behavioral support (e.g., CBT or peer-to-peer support group), and medication management (e.g., GLP-1 receptor agonists) for individuals presenting with overlapping UPFA and type 2 diabetes was described by Bennett et al. (109). According to this model, UPFA screening would be recommended routinely during check-ups in patients with diabetes, especially in patients with uncontrolled HbA1c, strong cravings for ultra-processed foods, or difficulties in adhering to dietary plans (109). Dietary counseling would support the patients to identify trigger foods and introduce abstinence to them, and blood glucose monitoring as a key part of maintenance management (109). Self-monitoring tools may be useful for these patients, along with goal clarification and support in moving toward their objectives. Interventions may also include encouraging engagement in activities with lower reward salience as alternatives to food for self-care, enhancing motivation to follow structured routines and pre-planned meals, and facilitating changes in the food environment (109).

### 4.1 Limits of the model and directions for further research

The stepped model proposed here needs to be confirmed by clinical studies, as it contains many elements extrapolated from other EDs, SUDs, and metabolic disorders. This model is not based on a systematic literature review; therefore, relevant information might have been missed. Also, interventions such as psychotherapy or pharmacotherapy need to be strictly individualised, based on a careful risk/benefit evaluation. Future research should prioritize construct validation and mechanism-based clarification before reaching the necessary clarification of the UPFA’s nosographic status. Comparative studies examining UPFA symptoms across populations with BED, obesity, and SUDs are needed to determine the incremental validity and clinical utility of this condition. Experimental models focused on reward learning, inhibitory control, and stress responsivity may help identify neurobehavioral profiles associated with severe dysregulation of ultra-processed food–related behaviors. Additionally, stratified treatment trials could examine whether individuals with prominent reward-driven eating patterns show differential responses to pharmacological or psychotherapeutic interventions, while carefully accounting for psychiatric and metabolic comorbidities. Such a cautious, mechanism-oriented research agenda may clarify whether UPFA reflects a distinct clinical phenotype, a transdiagnostic specifier, or a dimensional expression of existing disorders.

## 5 Conclusion

UPFA is a paradoxical condition from a historical perspective, because although it has a quite long history in the literature, scientific research in this domain has only been around for a few decades. Due to this paradox, the therapeutic recommendations for patients with UPFA remain speculative. A comprehensive conceptualization of UPFA that transcends the SUD–BA dichotomy was adopted as the basis for a multidimensional, mechanism-based understanding of this condition, emphasizing directions for future research A stepped model that integrates the RDoC core dimensions would serve not only to orient the mental health specialist in case management, but also to direct research focused on the pathophysiology of this condition. Using objective outcomes to monitor these patients’ evolution is considered important for validating the efficacy of each therapeutic intervention recommended. A multidisciplinary team comprising mental health specialists, endocrinologists, and nutritionists is a core component of case management for patients with UPFA. This perspective is expected to increase physicians’ and psychologists’ awareness of the complex, multidimensional nature of UPFA.

## Data Availability

The original contributions presented in the study are included in the article/supplementary material, further inquiries can be directed to the corresponding author.
